# Determination and Removal of Potentially Toxic Elements by *Phragmites australis* (Cav.) Trin. ex Steud. (Poaceae) in the Valles River, San Luis Potosí (Central Mexico)

**DOI:** 10.3390/plants14010033

**Published:** 2024-12-26

**Authors:** José Angel Montes-Rocha, Rocío del Carmen Diaz-Torres, Angel Josabad Alonso-Castro, César Arturo Ilizaliturri-Hernández, Leticia Carrizales-Yáñez, Candy Carranza-Álvarez

**Affiliations:** 1 Postgraduate in Chemical Sciences, Autonomous University of San Luis Potosí, San Luis Potosí 78210, Mexico; angel.montes@uaslp.mx; 2 School of Professional Studies Huasteca Zone, Autonomous University of San Luis Potosí, Ciudad Valles, San Luis Potosí 79060, Mexico; rocio.diaz@uaslp.mx; 3 Division of Natural and Exact Sciences, University of Guanajuato, Guanajuato 36000, Mexico; angeljosabad@ugto.mx; 4 Coordination for Innovation and Application of Science and Technology CIACYT-Faculty of Medicine, Autonomous University of San Luis Potosí, San Luis Potosí 78210, Mexico; cesar.ilizaliturri@uaslp.mx (C.A.I.-H.); letcay@uaslp.mx (L.C.-Y.)

**Keywords:** potentially toxic elements, water pollution, phytoremediation, *P. australis*

## Abstract

The contamination of rivers by potentially toxic elements (PTEs) is a problem of global importance. The Valles River is Ciudad Valles’ (Central Mexico) main source of drinking water. During the four seasons of the year, water samples (n = 6), sediment samples (n = 6), and *Phragmites australis* plants (n = 10) were taken from three study sites selected based on the presence of anthropogenic activities in the Valles River. A graphite atomic absorption spectrophotometer estimated elements in the water, and an energy-dispersive X-ray fluorescence spectrometer quantified elements in sediments and plant samples. *Phragmites australis* accumulated metal(loid)s mainly in the roots during all seasons of the year. Water samples from all sites recorded PTEs (As, Pb, Cd, and Hg), with primary sources identified as the sugar industry, urban and industrial wastewater, and the combustion of fossil fuels. Sediment samples showed concentrations of Hg, Mn, Ni, Zn, Pb, V, Cu, Cr, and Cd, attributed to agricultural practices, industrial activity, and urbanization. *P. australis* is an alternative for in situ phytoremediation because this macrophyte can bioaccumulate different elements in its roots, such as Mn, Rb, V, Sr, Cu, Zn, Pb, Ni, and As.

## 1. Introduction

Water is an essential resource for plants, animals, and humans. Lotic water bodies, such as rivers, are vital for the regional economy and ecological balance [1]. However, anthropogenic activities impact the quantity and quality of water by contaminating water bodies and soil, leading to an increase in pollution, a major global concern.

Potentially toxic elements (PTEs) are released into the environment by human activities like burning fossil fuels, mining and smelting metals, using chemical fertilizers and pesticides, irrigation with wastewater, and dumping industrial waste [2]. Once incorporated into the environment, PTEs can reach surfaces and groundwater and enter the food chain through their absorption by plants. The accumulation of PTEs can also occur in sediments [3]. Different factors affect the availability of PTEs, such as pH, redox potential, organic matter, clay, and oxide minerals. In solution, the reduction in the concentration of PTEs is mediated by the increase in pH levels caused by the complexation of metals with functional groups of organic matter and oxides that affect the elements’ solubility [2]. In soil and sediments, the redox potential affects the transformation, solubility, and absorption of PTEs by plants. The solubility of potentially toxic elements with large oxidation states is low, and their mobility becomes limited when they precipitate, thereby reducing their absorption by plants. Due to their high cation exchange capacity, organic matter, clay, or oxides can reduce the availability of PTEs. Moreover, humic substances can bond with some PTEs, turning them into non-exchangeable elements [4].

Sediments are classified as the main receptacles of PTEs that are present in surface waters [1,5,6]. Different chemical forms of these metal(loid)s can bind to sediments or mobilize to water under specific chemical and physicochemical conditions. The chemical interactions between aquatic systems and PTEs are complex, causing environmental changes at different levels of the food chain. The complexes formed with PTEs in sediments and water depend on changes in pH, potential redox, chemical species, and temperature [7]. Therefore, due to their adsorption capacity, sediments can minimize any effect of PTEs and may release PTEs into the water when their physical and chemical conditions change [5].

The Valles River basin is located mainly in the municipalities of Ciudad Valles and El Naranjo, in San Luis Potosi state, and Nuevo Morelos, in Tamaulipas state, in Mexico. It has an area of 3178.71 km^2^ and is the main water source for Ciudad Valles and the surrounding region. Over time, the river basin has supported various hydraulic activities, including hydroelectric power generation, agriculture (particularly sugarcane cultivation), livestock farming, agro-industrial factories, and urban supply [8]. However, the Valles River basin faces several problems, including drought, illegal dumping of domestic and industrial wastewater, leaching of agrochemicals, and the spread of invasive plant species such as *Typha* spp. and *Phragmites* spp. Various remediation methodologies have been developed to address PTE contamination, such as chemical precipitation, adsorption, electrosorption, electrochemical adsorption, ion exchange, adsorbents, and biological or nanotechnological approaches [9]. Phytoremediation, a non-intrusive, cost-effective, and socially accepted method for cleaning contaminated sites, uses plants to remove pollutants withstanding the damage caused by PTEs [10,11,12]. In aquatic environments, macrophytes are particularly effective at inactivating and accumulating PTEs in their shoots and roots [13,14]. The selection of macrophytes for phytoremediation is based on their robustness and ability to tolerate metal(loid) toxicity. Aquatic species like *Limnocharis flava*, *Typha* spp., *Scirpus* spp., *Spartina* spp., and *Phragmites* spp. are recommended for remediating water bodies affected by metal(loid) contamination [15,16]. Notably, the *Phragmites* genus is a natural alternative for treating wastewater contaminated with metals, playing a crucial role in water purification [17]. *Phragmites australis* (common reed, Poaceae) is a cosmopolitan, tall, perennial, helophytic grass with extensive rhizome systems and annual shoots up to 5 m long [18]. Due to its capacity to grow in extreme conditions and its associations with microorganisms and biochemical adaptations, this macrophyte has become the preferred plant system for improving wastewater quality [19]. This study assessed the presence of PTEs and analyzed the physicochemical properties of water and sediments from the Valles River. This study also evaluated the ability of *Phragmites australis* to accumulate several elements. Data collection occurred across different seasons to understand the river’s hydrodynamic conditions.

## 2. Materials and Methods

### 2.1. Flowchart of the Procedure

**Figure 1:**
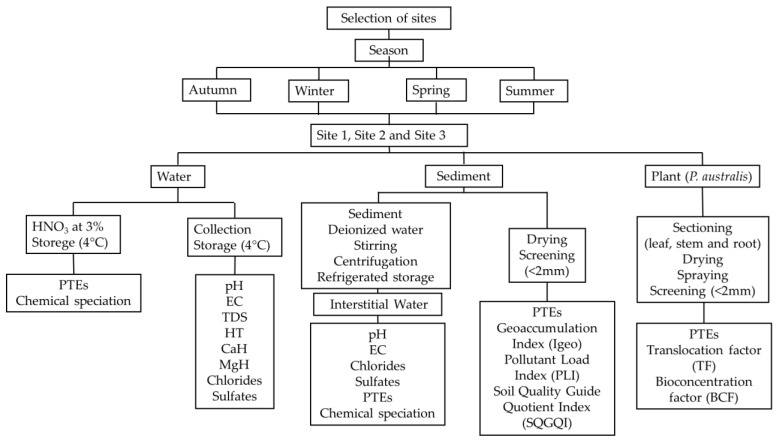
Experimental design and workflow of this study.

For the evaluation of PTEs and other elements, surface water, sediment, and plant samples were taken. The water samples were analyzed using graphite atomic absorption, and the sediment and plant samples were analyzed using an X-ray energy dispersive fluorescence spectrometer. The sediment results were used to determine the geoaccumulation index (Igeo), the pollution load index (PLI), and the soil quality guide quotient index (SQGQI). The translocation factor and the bioconcentration factor were determined with the plant results. The physicochemical properties were evaluated in surface water and interstitial water. And chemical speciation modeling was performed with the results of surface and interstitial water (Figure 1).

### 2.2. Study Area

**Figure 2:**
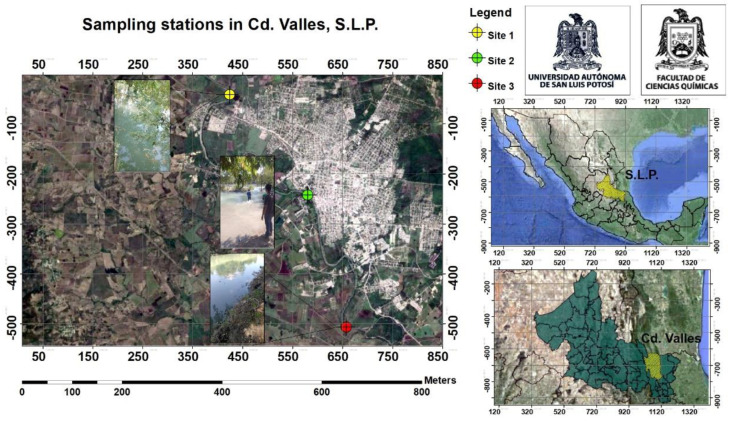
Location of sediment and plant sampling sites.

The Valles River watershed is located in Ciudad Valles, S.L.P., Mexico, in the region known as Huasteca Potosina (Figure 2). This river is important for both local biodiversity and human activities, as it is the only source of potable water for consumption, agriculture, and industry. This river originates in the Sierra Madre Oriental and flows eastward through agricultural, industrial, urban, and rural areas. The Valles River basin has a warm, humid climate with abundant rainfall. Table 1 displays the location and description of the three sampling sites.

**Table 1:** Location and description of sampling sites.

Site	Geolocation	Site Description
Site 1	22°01′14.0″ N 99°02′57.5″ W	The sugar mill is located in this area and is surrounded by agricultural activities of the sugar industry (agrochemical leaching).
Site 2	21°59′11.2″ N 99°01′16.6″ W	Urban area (leaching of domestic wastewater and commercial wastewater).
Site 3	21°56′38.5″ N 99°00′24.9″ W	Area impacted by urban wastewater, commercial wastewater, and agrochemical leaching (agro–sugar industry).

### 2.3. Sampling

One liter of water samples (n = 6 from each study site) was collected in polypropylene bottles previously washed with 10% nitric acid. The samples were acidified with 3% nitric acid and stored at 4 °C for their preservation during transfer. A second liter of water sample (n = 6 from each study site) was collected, transported in cold storage, and kept at 4 °C for analyzing physicochemical properties.

Approximately 500 g of sediment samples (n = 6 from each study site) were collected using plastic shovels, stored in plastic bags, and transported to the laboratory. The sampling procedure for each type of analysis adhered to the Official Mexican Standard NOM-014-SSA1-1993 (SSA, 1994), which outlines sanitary procedures for sampling water intended for human use and consumption in public and private water supplies. The sediment samples were dried in an oven (Lindberg/BlueM) for 48 h at 60 °C, sieved to <2 mm, and stored for further analysis.

Plant samples (n = 10 from each sampling site) were collected in plastic bags, transported to the laboratory, pre-washed with EDTA (0.5 M), sectioned into roots–rhizomes, stems, and leaves, and dried in an oven (Lindberg/BlueM) at 50 °C for four days. The dried plant material was processed in an analytical mill (IKA^®^ WERKE, Staufen, Germany) to a fine powder of less than 2 mm, and the pulverized samples were stored for future analysis.

### 2.4. Quality Control

#### 2.4.1. Laboratory Equipment

The multiparameter probe, model Groline HI9814 (HANNA Instruments, Woonsocket, RI, USA), was calibrated with a certified standard (QuickCal, HI50036, HANNA Instruments, Woonsocket, RI, USA) at each sampling site.

The PerkinElmer graphite atomic absorption spectrophotometer, model PinAAcle 900T, equipped with a furnace autosampler (model AS900), was used to determine Pb, As, and Cd in water. Standard Reference Material NIST 1643f was used as a positive control, with recovery percentages of Pb (98%), As (92%), Cd (97%), Cr (97%), and Mn (98%). The reference material was determined every 25 samples.

The PerkinElmer atomic absorption spectrophotometer, model AAnalyst 100, with a flow injection system (model FIAS 100), was used to determine Hg in water using the Standard Reference Material NIST 1641e as a positive control with a recovery percentage of 98%. The reference material was determined every 25 samples.

Energy-dispersive X-ray fluorescence spectrometer (EDX-7000/8000/8100, Shimadzu, Kyoto, Japan), Kodak Standard Reference Material, 15087 for sediment, and NIST, 1545 for plants were used as controls.

#### 2.4.2. Reagents

Silver nitrate (AgNO_3_) ≥99.0%; ethylenediaminetetraacetic acid (EDTA) ((HOOCCH_2_)_2_NCH_2_CH_2_N(CH_2_COOH)_2_); Eriochrome^®^ Black T ACS (indicator grade); murexide (C_8_H_8_N_6_O_6_) (indicator grade); potassium chromate (K_2_CrO_4_); barium chloride (BaCl_2_); sodium chloride (NaCl) ≥99.0%; chloric acid (HCl); glycerol (HOCH_2_CH(OH)CH_2_OH); and ethanol ≥95.0% were obtained from Sigma-Aldrich (St. Louis, MO, USA).

### 2.5. Determinations in Surface and Interstitial Water

#### 2.5.1. Pretreatment

Surface water was used directly from the river. For the interstitial water, 25 g of each sediment sample were placed in 50 mL conical tubes, and 50 mL of deionized water was added. The tubes were placed on a plate shaker for 24 h, centrifuged at 3000 rpm for 15 min, and the supernatant was separated. Subsequently, the supernatant was filtered through 11 µm Whatman filter paper. The filtrate was placed in 15 mL conical tubes and stored at 4 °C until use [20,21,22]. The technique was modified by adding deionized water to extract only the exchangeable fraction.

#### 2.5.2. Determination of pH, Electrical Conductivity (EC), Total Dissolved Solids (TDS), and Temperature (°C)

Quantification of pH, electrical conductivity (EC), total dissolved solids (TDS), and temperature (°C) was performed using a multiparameter probe (Groline HI9814, HANNA Instruments, Woonsocket, RI, USA), which was previously calibrated. Measurements were taken in situ by activating the equipment and immersing the probe in the water body. The readings were then recorded for analysis.

#### 2.5.3. Chloride Determination

For surface water, the technique described in the Mexican Natural Water Standard (NOM) AA-073-SCFI-2001 [23] was used to determine chloride content. The method is based on titration with silver nitrate using potassium chromate as an indicator. Silver reacts with the chlorides in the solution to form silver chloride. Subsequently, the chromate begins to precipitate, leading to the formation of silver chloride.

The chloride concentration in the interstitial water was estimated according to the procedure described by national legislation [22]. Five milliliters of the saturation extract were mixed with 15 mL of deionized water, and 4 drops of potassium chromate were added as an indicator. The solution was then titrated with 0.025 N AgNO_3_ until a color change from yellow to brick red occurred. The chloride concentration was calculated using the following equation:

(1) $${m\;mol}_{\left(-\right)}L^{-1}=\frac{{mL\;de\;AgNO}_{3}\times {N\;de\;AgNO}_{3}\times 1000}{mL\;aliquot}$$

#### 2.5.4. Sulfate Determination

For surface water, the method set out in Mexican Official Standard (NOM) AA-074-SCFI-2014 [24], was used. This standard describes how to measure sulfate ions in natural waters using turbidimetry. The method is based on the precipitation of sulfate ions with barium chloride in an acid medium, forming barium sulfate crystals.

The sulfate concentration in the interstitial water was estimated following the national legislation protocol [22]. Ten milliliters of saturation extract were mixed with 100 mL of deionized water, followed by the addition of 5 mL of a conditioning solution (containing 75 g NaCl, 30 mL HCl, 50 mL glycerol, and 100 mL of 96% ethanol in 1 L of deionized water) and 0.2 g barium chloride. The samples were shaken for 60 s, and absorbance was measured at 340 nm. Simultaneously, a calibration curve (0.0, 0.1, 0.2, 0.3, 0.4, and 0.5 meq/L SO_4_^2−^) was prepared following the same procedure for the samples. The sulfate concentration was then calculated using the following equation:

(2) $${m\;mol\;L}^{-1}{SO}_{4}^{2-}=a\times 10\times d$$

where a is the calculated C mol L on the curve, 10 is the curve’s dilution factor, and d represents the sample’s dilution factor.

#### 2.5.5. Determination of Total Hardness (TH), Calcium Hardness (CaH), and Magnesium Hardness (MgH)

The Mexican Official Standard (NOM) AA-72-1981 [25] describes a technique for determining total hardness in surface water, based on the formation of complexes by the disodium salt of ethylenediaminetetraacetic acid with calcium and magnesium ions. Erythrochrome black was used as an indicator for TH and murexide for CaH. The difference between TH and CaH yielded the MgH value. This determination did not apply to interstitial water.

#### 2.5.6. Determination of PTEs (Pb, As, Cd, and Hg)

Surface and interstitial water samples were thawed at room temperature before digestion in a Lab Tech autoclave at 121 °C for 30 min. Subsequently, the samples were filtered (Whatman 11 µm). A PerkinElmer graphite atomic absorption spectrophotometer, model PinAAcle 900T, with a furnace autosampler (model AS900), was used to measure the amounts of arsenic (As), lead (Pb), cadmium (Cd), mercury (Hg), chromium (Cr), and manganese (Mn). Mercury (Hg) concentrations were determined using the cold vapor method with a PerkinElmer atomic absorption spectrophotometer, model AAnalyst 100, with a flow injection system (model FIAS 100).

For quality control, certified standards were utilized to create standard curves, and enriched controls with known concentrations of the target elements were employed. Standard Reference Material NIST 1643f was used for the enriched controls of As, Pb, and Cd, and Standard Reference Material NIST 1641e was utilized for Hg in trace elements in water. Recovery percentages for the PTEs from the analysis were as follows: As (92%), Pb (98%), Cd (97%), Hg (98%), Cr (97%), and Mn (98%), ensuring adequate quality control throughout the process.

#### 2.5.7. Chemical Speciation

Chemical speciation was performed for surface and interstitial water using ChemEQL-3.1 software (Beat Müller, Swiss Federal Institute of Environmental Science and Technology, CH-6047 Kastanienbaum, Switzerland), considering the pH conditions of the Valles River.

### 2.6. Determination of PTEs in Sediment and Plant

An energy-dispersive X-ray fluorescence spectrometer (EDX-7000/8000/8100, Shimadzu, Japan) determined the concentrations of Pb, Hg, Ni, Co, Mn, Cu, Cd, Zn, Cr, V, and As in sediment and plant samples (which were previously sectioned, dried, crushed, and sieved). Two grams of each sediment or plant sample were placed on a 6-micron-thick mylar film with a diameter of 64 mm, housed in a 10 mL polypropylene sample holder (31 mm open-end X-cell), and mounted inside the spectrometer. The elemental analysis was performed in an air-based atmosphere using a 10 mm diameter collimator and an acquisition time of 120 s, operating at 50 kV and 1000 μA, controlled via the PCEDX-Navi ver. 2.01 software.

For quality control, calibration was carried out using standard reference materials for metals in sediments (Kodak, 15087) and plants (NIST, 1547). The accuracy and precision of the EDX measurements were validated, with a difference of less than 2% (% diff) and a relative standard error (RSE) below 9%. The concentrations obtained for the sediment and plant samples were expressed in milligrams per kilogram of dry weight (mg/kg s.w.). Detection limits (mg/kg) for the analyzed elements were as follows: Ag (1.0), Al (10.0), As (0.1), Ba (1.0), Cd (1.0), Co (1.0), Cr (1.0), Cu (1.0), Fe (1.0), Hg (0.1), K (1.0), Mn (1.0), Mo (0.1), Ni (1.0), P (10.0), Pb (0.1), Sb (1.0), Se (0.1), Sn (1.0), Sr (0.1), Ti (1.0), Tl (0.1), V (1.0), and Zn (0.1).

### 2.7. Sediment Indexes

#### 2.7.1. Geoaccumulation Index (Igeo)

The geoaccumulation index (I_geo_), a quantitative measurement used in exploratory factor analysis (EFA) to assess contamination levels in sediments, is calculated using the following equation:

(3) $$I_{geo}={Log}_{2}^{{(C}_{n}/{1.5B}_{n})}$$

Cn is the amount of the element that was found in the sediment sample, Bn is the potentially toxic element’s natural background value, and 1.5 is a factor that considers changes in background values that happen over time. The classes of the value of I_geo_ are as follows: <0: not contaminated, 0–1: not contaminated to moderately contaminated, 1–2: moderately contaminated, 2–3: moderately to heavily contaminated, 3–4: heavily contaminated, 4–5: heavily to extremely contaminated, and >5: extremely contaminated [26].

#### 2.7.2. Pollution Load Index (PLI)

The Pollution Load Index (PLI) provides straightforward yet comprehensive information and assesses contamination levels by PTEs: Pb, Hg, Ni, Co, Mn, Cu, Cd, Zn, Rb, Ba, Cr, and As. The PLI is calculated using the following equation:

(4) $$CF=\frac{C_{metal}}{C_{baselin}}$$

(5) $$PLI=\sqrt[{n}]{\left({CF}_{1}\times {CF}_{2}\times \ldots \times {CF}_{n}\right)}$$

The contamination factor (CF) is determined by comparing the environmental concentration of each metal (Cmetal) with its background value (Cbaseline). The PLI is classified into four categories: uncontaminated (PLI < 1), moderately contaminated (1 < PLI < 2), intensely contaminated (2 < PLI < 3), and extremely contaminated (PLI > 3) [27].

#### 2.7.3. Soil Quality Guide Quotient Index (SQGQI)

For the determination of the SQG, the following PTEs were considered: Pb, Hg, Ni, Co, Mn, Cu, Cd, Zn, Rb, Ba, Cr, and As. They were calculated using the following equation:

(6) $$SQGQI=\sum_{i=1}^{n}{(CE}_{i}/{CR}_{i}){\times n}^{-1}$$

The NOAA SQuiRT tables for inorganic substances in sediments provide the reference concentration, CR, whereas CE represents the ambient concentration of PTEs. The variable n refers to the number of elements evaluated. The average ratio of potential exposure to PTEs, known as the Sediment Quality Guideline Quotient Index (SQGQI), indicates a level of no expected adverse effects. If the SQGQI exceeds 1, adverse effects may occur. This index is particularly valuable because it assesses the chemical contamination level of the samples, helping classify them as either toxic or non-toxic [28].

### 2.8. Indexes for Plants

#### 2.8.1. Translocation Factor (TF)

The translocation factor (TF) was calculated for the plants sampled across the three sampling sites. A TF greater than 1 indicates that the plant accumulates metals in its aerial parts, whereas a TF less than 1 suggests that heavy metals primarily accumulate in the underground parts. The TF is determined using the following equation [29]:

(7) $$TF=\frac{\left(Metal\;concentration\;in\;sheet\right)}{\left(Metal\;concentration\;in\;root-rhizome\right)}$$

#### 2.8.2. Bioconcentration Factor (BCF)

The bioconcentration factor (BCF) was calculated using the following equation:

(8) BCF=CP CS

CP refers to the metal concentration in the plant (root–rhizome and leaf), whereas CS denotes the metal concentration in the sediment. The BCF was determined for root–rhizome and leaf. A BCF greater than 1 indicates that the plant is efficient at accumulating metals [30].

### 2.9. Statistical Analysis

#### 2.9.1. Univariate Analysis

Using 1% and 5% significance levels, the Kruskal–Wallis test looked at whether there were significant differences in the physicochemical properties and exploratory factor analysis (EFA) of water and sediment based on where the samples were taken and the time of year. Additionally, the test assessed the levels of elements in plants according to sampling site, season, and organ type. Statistical analyses were conducted using GraphPad Prism (version 8.0.2) and STATISTICA 10.0.

#### 2.9.2. Multivariate Analysis

Basic descriptive statistics calculated the median, minimum, and maximum values. Tables were created for each sampling site, categorizing results based on sediment or plant samples. Separate tables were generated for each sampling site and plant part using STATISTICA 10.0. A permutation multivariate analysis of variance (PERMANOVA) compared physicochemical properties and PTEs. The model considered three factors: sampling site (three levels: sampling sites 1, 2, and 3), season (four levels: autumn, winter, spring, and summer), and plant organ (three levels: leaf, stem, and root). A total of 9999 permutations of residuals were performed under a reduced model, using a three-way sum of squares to account for unbalanced data.

Subsequently, a multiple-comparison analysis compared sampling sites, seasons, and plant organs. SIMPER found the elements and physicochemical properties that were most important in explaining the differences between sampling sites, seasons, and plant parts. Results from the PERMANOVA were visually represented using Canonical Analysis of Principal Coordinates (CAP). All multivariate analyses were performed using PRIMER 6 statistical software, with statistical significance evaluated at the 1% and 5% levels.

## 3. Results

### 3.1. Univariate Analysis of Surface Water by Sampling Site

Sampling site 1 recorded (*p* < 0.05) pH variations between 6.92 and 7.84 and elevated sulfate concentrations ranging from 13.95 to 30.72 mg/L. Sampling site 2 recorded the highest chloride concentration (3.22–28.79 mg/L) (*p* < 0.05), and sampling site 3 recorded a TDS concentration of 0.04–0.10 µg/L (*p* < 0.05). Concentrations during the four seasons of the year were recorded for EC (0.05–0.11 mS), temperature (20.3–32.8 °C), TH (128.0–660.0 mg/L), CaH (26.0–452.0 mg/L), MgH (28.0–526.0 mg/L), Pb (0.001–0.01 mg/L), Cd (6.0 × 10^−3^–0.001 mg/L), and As (2.0 × 10^−5^–0.05), showing no significant differences according to sampling site. Cr and Mn were not detectable, and Hg was only detected at one sampling site.

### 3.2. Multivariate Analysis of Surface Water by Sampling Site

**Figure 3:**
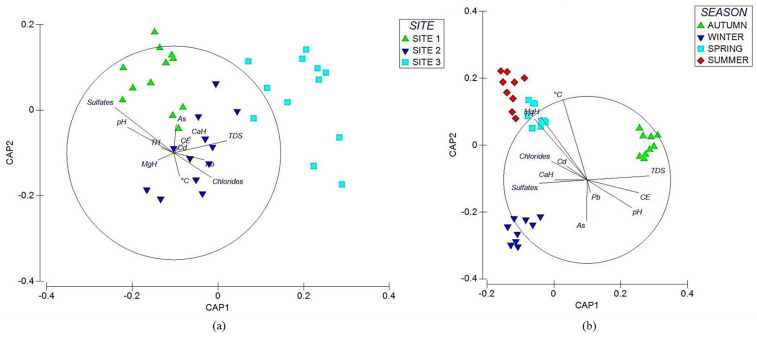
Canonical principal coordinate analysis (CAP) of physicochemical properties and potentially toxic elements (PTEs) in water. The analysis shows the variability of the data and differences between each site and season. The direction of the vectors indicates the increase in physicochemical properties and PTEs at the sites. The graph displays (**a**) the CAP by site and (**b**) the CAP by season.

Sampling sites, physicochemical properties, and elements showed significant differences (Pseudo-F = 5.46, *p* < 0.01). Each sampling site showed a different response to each variable. A canonical analysis of principal coordinates (CAP) explained the behavior of each variable and sampling site (Figure 3a). Table 2 shows the pairwise comparisons (*t*-value), the contribution of physicochemical properties, and pairwise elements (50% dissimilarity). The SIMPER test showed that the variables contributing to dissimilarity at sampling sites 1 and 2 were As, sulfates, temperature, and MgH. TDS, Cd, pH, Pb, and As were the variables contributing to dissimilarity at sampling sites 1 and 3, whereas TDS, Cd, EC, and Pb were the variables contributing to dissimilarity between sampling sites 2 and 3 (Table 2).

### 3.3. Univariate Analysis of Surface Water by Season

The different variables assessed each season showed significant differences. Autumn recorded pH (7.7–7.9) and EC (0.110–0.115 mS) values, and winter registered As concentrations (9.4 × 10^−3^–0.05 mg/L). Spring recorded chlorides (11.91–28.79 mg/L), CaH (76.0–384.0 mg/L), MgH (192.0–478.0 mg/L), and TDS (0.05–0.1 µg/L) concentrations (*p* < 0.01). Summer showed levels of temperature (30.70–32.80 °C), sulfates (7.08–30.72 mg/L), and TH (430–660 mg/L) (*p* < 0.01). The levels of Pb (2.0 × 10^−5^–0.01 mg/L) and Cd (5.8 × 10^−3^–0.001 mg/L) assessed in each season showed no significant differences.

### 3.4. Multivariate Analysis of Surface Water by Season

Physicochemical properties and some elements showed differences between seasons. The response of each variable was different in each season. A canonical analysis of principal coordinates (CAP) recorded the behavior of the variables (Figure 3b). Table 3 shows the pairwise comparisons (*t*-value) and the contributions of physicochemical properties and elements by season (50% dissimilarity). The main variables that favored dissimilarity between each of the seasons were As, chlorides, EC, and pH (autumn vs. winter); EC, TH, CaH, and MgH (autumn vs. spring); and TH, MgH, Pb, Cd, and pH (autumn vs. summer) (Table 2).

**Table 3:** Cumulative percentage of physicochemical properties and PTEs in water by SIMPER analysis by season of the year.

Season	*t*-Value (Cumulative Percentage)
Autumn vs. winter	4.0, (50%) * (As, Chlorides, CE, pH)
Autumn vs. spring	5.9, (50%) * (CE, TH, DCa, DMg)
Autumn vs. summer	4.4, (50%) * (TH, DMg, Pb, Cd, pH)
Winter vs. spring	3.5, (50%) * (TDS, As, temperature, DMg)
Winter vs. summer	3.1, (50%) * (°C, As, DMg, Cd)
Spring vs. summer	2.1, (50%) * (TDS, Cd, Sulfates, Pb)

PERMANOVA analysis *t*-value, (*) significant differences (*p* < 0.01).

### 3.5. Univariate Analysis of Interstitial Water by Site

Sampling site 1 showed a higher pH (6.5–7.3) than the other sites (*p* < 0.005). And site 3 registered elevated concentrations of chlorides (1.2–18.0 mmol L^−1^), sulfates (7.6–26.1 mmol L^−1^), and As (0.003–0.01 mg/L) with respect to the other sites (*p* < 0.005). EC (0.04–0.5 mS), Pb (2.0 × 10^−3^–0.009 mg/L), and Cd (5.0 × 10^−3^–0.001 mg/L) showed no significant differences; however, concentrations were recorded throughout the four seasons.

### 3.6. Multivariate Analysis of Interstitial Water by Site

**Figure 4:**
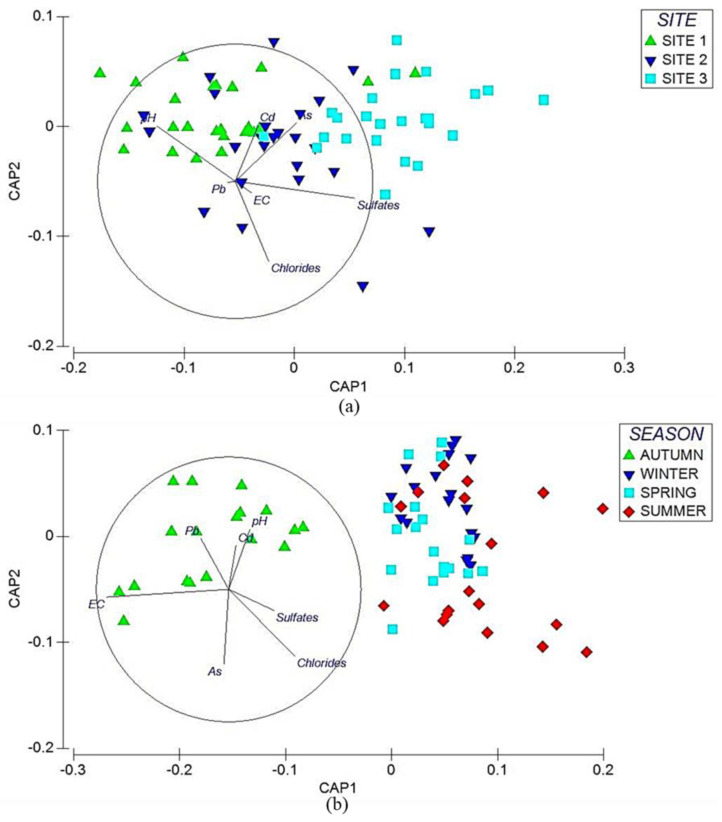
Canonical principal coordinate analysis (CAP) of physicochemical properties and potentially toxic elements (PTEs) in interstitial water. The analysis shows the variability of the data and differences between each site and season. The direction of the vectors indicates the increase in physicochemical properties and PTEs at the sites. The graph displays (**a**) the CAP by site and (**b**) the CAP by season.

The physicochemical properties and elements per site showed significant differences (Pseudo-*F* = 4.56, *p* < 0.01). Each site yielded a unique response for each variable. Canonical principal chord analysis explains the behavior of each variable per site (Figure 4a). Table 4 shows the pairwise comparisons (*t*-value), the contribution of physicochemical properties, and pairwise elements (50% dissimilarity). The SIMPER test for dissimilarity showed that the variables contributing to the dissimilarity between sites 1 and 2 were pH, chlorides, and Cd. Sulfates, pH, and Cd were the variables that presented dissimilarity at sites 1 and 3, whereas chlorides, Pb, and EC were the variables that contributed to the dissimilarity between sampling points 2 and 3 (Table 4).

### 3.7. Univariate Analysis of Interstitial Water by Season

The winter (0.08–0.09 mS) and spring (0.08–0.09 mS) seasons recorded elevated EC with respect to the other seasons (*p* < 0.05). And chloride (1.7–18.0 mmol L^−1^) and sulfate (6.7–19.7 mmol L^−1^) concentrations were identified in summer. The pH (6.0–7.3), Pb (2.0 × 10^−3^–0.009 mg/L), Cd (5.0 × 10^−3^–0.001mg/L), and As (9.8 × 10^−5^–0.01 mg/L) showed no significant differences.

### 3.8. Multivariate Analysis of Interstitial Water by Season

Seasonal differences were observed in the physicochemical properties and certain elements, with the exception of autumn compared to summer. Each variable behaved differently, and to understand the behavior, a canonical principal coordinate analysis (CAP) was performed (Figure 4b). Table 5 shows the pairwise comparisons (*t*-value) and the contributions of physicochemical properties and elements by season (50% dissimilarity). The variables that showed dissimilarity between each of the seasons were EC, Pb, and Cd (fall vs. winter); EC, Cd, and Pb (fall vs. spring); Pb, Cd, and sulfates (winter vs. spring); chlorides, As, and pH (winter vs. summer); and finally, chlorides, As, and sulfates (spring vs. summer).

### 3.9. Univariate Analysis of Sediment by Sampling Site

Sampling site 1 recorded significant differences in the concentrations (mg/kg) of Ni (44.2–207.7) and Hg (57.6–84.8) (*p* < 0.05). At sampling site 2, concentrations (mg/kg) of Cu (71.8–165.2), Zn (42.6–434.2), Pb (15.0–45.6), and V (33.2–152.9) were recorded (*p* < 0.05). Zn concentrations ranged from 42.6 to 434.2 mg/kg at sampling site 3 (*p* < 0.05). There were no significant differences between sampling sites for Mn (113.1–482.3 mg/kg), Cr (29.80–74.80 mg/kg), As (5.8–44.5 mg/kg), and Cd (12.40–23.20 mg/kg). The sediment samples did not contain any Co content.

### 3.10. Multivariate Analysis of Sediment by Sampling Site

**Figure 5:**
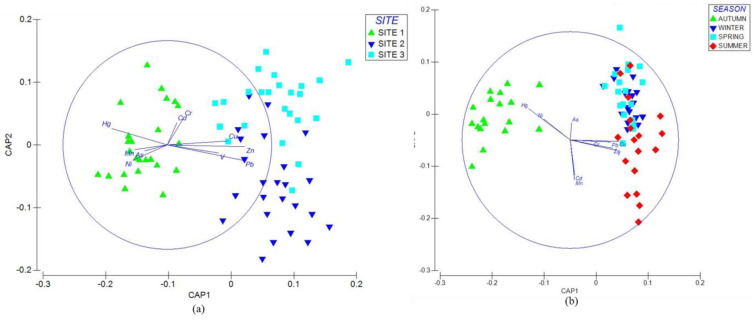
Canonical principal coordinate (CAP) analysis of physicochemical properties and elements in sediment. The analysis showed the variability of the data and the differences between each site and season. The direction of the vectors indicates the increase in physicochemical and chemical element properties at the sampling sites. The vectors show (**a**) CAP per sampling site and (**b**) CAP per season.

Elements found in sampling sites showed significant differences (Pseudo-*F* = 7.53, *p* < 0.01). Each sampling site was characterized by a different response. A canonical analysis of principal coordinates (CAP) provided information on the behavior of the variables in the sampling sites (Figure 5a). Table 6 shows the comparisons between pairs (*t*-value) and the contribution of the physicochemical properties and elements by pairs (50% dissimilarity). The variables contributing to the dissimilarity between sampling sites 1 and 2 were Zn, Pb, V, Cu, Hg, and Mn. The variables providing the dissimilarity between sampling sites 1 and 3 were sulfates, Ni, Pb, Mn, Cr, and Cd. The variables contributing to the dissimilarity between sampling sites 2 and 3 were V, Cu, Zn, As, and Cd.

### 3.11. Univariate Analysis of Sediment by Season

Spring and summer recorded the highest Cu concentrations (64.4–165.2 and 73.0–145.9 mg/kg) (*p* < 0.05). The variables V, Pb, Ni, Zn, Cd, Cr, and As did not show significant differences for each season. However, concentrations (mg/kg) of PTEs (V, 27.1–152.9; Pb, 10.0–45.6; Ni, 44.2–207.7; Zn, 37.7–573.1; Cd, 12.4–23.2; Cr, 29.8–74.8, and As, 5.8–44.5) were recorded for all four seasons.

### 3.12. Multivariate Analysis of Sediment by Season

Only autumn recorded significant differences in the variables (Pseudo-*F* = 4.12, *p* < 0.01). A canonical analysis of principal coordinates (CAP) evaluated the behavior of the variables (Figure 5b). Table 7 shows the pairwise comparisons (*t*-value) and the percentage contribution of PTEs in sediments from different seasons (50% dissimilarity). V, Ni, Cd, and Hg contributed to the dissimilarity of autumn vs. winter, whereas Pb, Cu, Zn, Cd, and Hg participated in the dissimilarity of autumn vs. spring. Cr, As, Hg, Pb, and Ni contributed to the dissimilarity of autumn vs. summer.

**Table 7:** Cumulative percentage of physicochemical properties and PTEs in sediment through SIMPER analysis by season of the year.

Season	*t*-Value (Cumulative Percentage)
Autumn vs. winter	2.3, (50%) * (V, Ni, Cd, Hg)
Autumn vs. spring	2.9, (50%) * (Pb, Cu, Zn, Cd, Hg)
Autumn vs. summer	3.0, (50%) * (Cr, As, Hg, Pb, Ni)

PERMANOVA analysis *t*-value, (*) significant differences (*p* < 0.01).

### 3.13. Pollution Rates

#### 3.13.1. Geoaccumulation Index (Igeo)

The Igeo results showed that the sampling sites 1, 2, and 3 had some degree of contamination (Table 8). Sampling site 1 recorded V, Mn, Co, and Pb concentrations as uncontaminated to moderately contaminated; Cr, Cu, and Zn levels were moderately contaminated; Ni concentrations were moderate to heavily contaminated; and As, Cd, and Hg levels were extremely contaminated. Sampling site 2 registered V, Cr, Mn, and Co levels as not contaminated to moderately contaminated, Cr and Pb levels as moderately contaminated, Ni and Cu as moderately to heavily contaminated, and Zn, As, Cd, and Hg concentrations as extremely contaminated. At sampling site 3, V, Mn, and Co concentrations ranged from not to moderately contaminated; Cr and Pb concentrations were moderately contaminated; Ni and Cu levels ranged from moderate to strongly contaminated; and Zn, As, Cd, and Hg levels were extremely contaminated.

#### 3.13.2. Pollution Load Index (PLI) and Soil Quality Guide Quotient Index (SQGQI)

**Figure 6:**
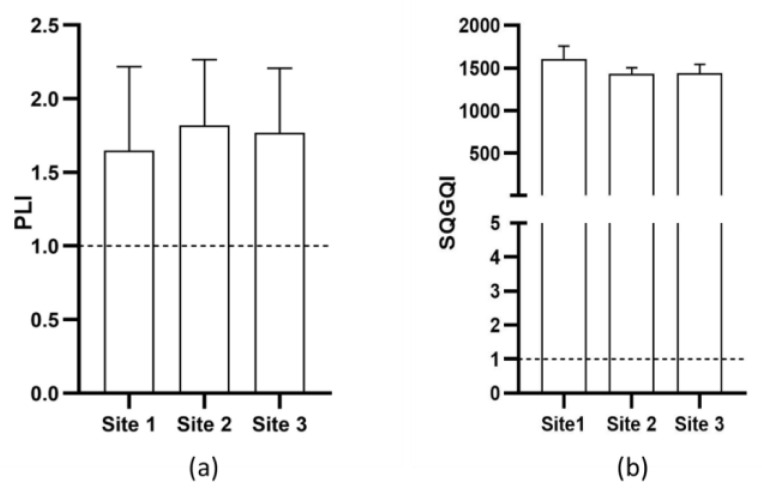
The EFA indices are presented by sites in the Valles River. (**a**) Pollution Load Index (PLI). (**b**) Soil Quality Guide Quotient Index (SQGQI). The diagram shows the median and interquartile range. Dashed lines represent the allowable limit.

Figure 6 shows the results of the PLI Index and the SQGQI Index in sampling sites 1, 2, and 3. Both indices were high in the three sampling sites. The values obtained for PLI and SQGQI at sampling sites 1 (1.64, 1605.93), 2 (1.81, 1431.72), and 3 (1.77, 1438.34) were above the threshold values. Therefore, these sampling sites presented some degree of affectation.

### 3.14. Univariate Analysis for P. australis and Sampling Site

The elements and sampling sites showed significant differences. Sampling site 1 recorded elevated concentrations (mg/kg) of Mn (19.6–576.6) and Ni (1.4–4.9) (*p* < 0.05). Sampling site 2 recorded elevated concentrations (mg/kg) of Mn (15.0–338.4) (*p* < 0.05), whereas sampling site 3 recorded elevated levels (mg/kg) of Cu (3.4–14.7), Zn (11.6–74.1), Pb (0.4–1.1), and Ni (1.4–6.0) (*p* < 0.05). Hg, Co, Cd, and Cr were not detected in plant samples.

### 3.15. Multivariate Analysis for P. australis and Sampling Site

**Figure 7:**
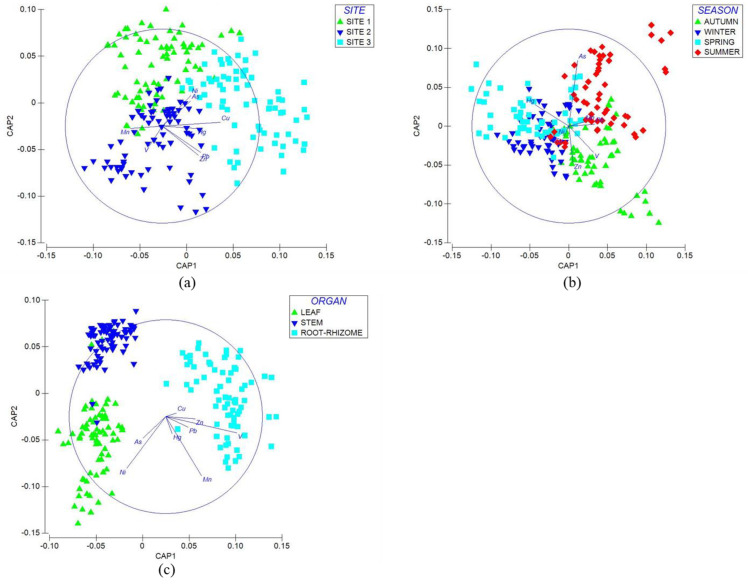
Canonical analysis of principal coordinates (CAP) of PTEs in plant samples. The analysis shows the variability of the data as well as the differences between factors (site, season, and organ). The direction of the vectors indicates the increase in plant PTEs per site. The vectors show an increase in (**a**) CAP per site, (**b**) CAP per season, and (**c**) CAP per organ.

The analysis showed significant differences (PERMANOVA, Pseudo-*F* = 53.72, *p* < 0.01) between sampling sites and the accumulation of the elements in the plant. Each site showed different variables, and each variable showed a different response. A canonical analysis of principal coordinates (CAP) analyzed the response of each variable for each sampling site (Figure 7a). Table 9 shows the comparisons between pairs (*t*-value) and the percentage contribution of EFAs in each sampling site (50% dissimilarity). Mn, V, and Ni participated in the dissimilarity between sampling sites 1 and 2, whereas Cu, Zn, and Pb affected the dissimilarity for sampling sites 1 and 3. Cu, Zn, and As contributed to the dissimilarity for sampling sites 2 and 3.

### 3.16. Univariate Analysis for P. australis and Sampling Season

In autumn, there were significant differences in the concentrations (mg/kg) of Zn (16.1–74.1), V (0.1–2.0), and Cu (3.3–5.2) (*p* < 0.05). The highest concentrations of Cu (2.6–14.7 mg/kg) were identified in the spring (*p* < 0.05). Statistical analysis revealed no significant differences for Mn, Ni, and Pb. But throughout the four seasons, concentrations (mg/kg) of Mn (8.0–338.4), Ni (1.2–6.0), and Pb (0.3–1.1) were recorded.

### 3.17. Multivariate Analysis for P. australis and Sampling Season

The analysis showed significant differences (PERMANOVA, Pseudo-*F* = 42.70, *p* < 0.01) between seasons and elements. A canonical principal coordinate analysis (CAP) identified which elements described each season (Figure 7b). Table 10 shows the pairwise comparisons (*t*-value) and the percentage contribution of elements between sampling sites. Each season showed a different response. However, there were no significant differences between seasons. When performing the canonical analysis of principal coordinates, Zn, V, and Pb contributed to the dissimilarity between autumn vs. winter, whereas Cu, Zn, and Ni favored the dissimilarity between autumn vs. spring. The elements that promoted the similarity between autumn vs. summer were Zn, Mn, and As. On the other hand, Cu, Ni, and Pb contributed to the dissimilarity in winter vs. spring. In winter and summer, Mn, As, and Pb enhanced the dissimilarity, whereas Cu, Mn, and Ni promoted the dissimilarity of spring and summer.

### 3.18. Univariate Analysis per Plant Organ in P. australis

There was a significant difference between plant organs when comparing the different elements. The leaves accumulated high concentrations of Ni (1.8–6.0 mg/kg) (*p* < 0.05). Roots–rhizomes recorded concentrations (mg/kg) of V (0.4–2.0), Zn (11.4–74.1), Mn (37.8–576.6), and Pb (0.3–1.0) (*p* < 0.05). There were no significant differences in Cu accumulation; however, the plant accumulated Cu concentrations in its organs (4.5–14.7 mg/kg).

### 3.19. Multivariate Analysis per Organ in P. australis

Significant differences existed (PERMANOVA, Pseudo-F = 235.75, *p* < 0.01) between the plant organ and each element. Each element’s response varied depending on the plant organ, and a canonical principal coordinate analysis (CAP) examined the behavior of the variables (Figure 7c). Table 11 shows the pairwise comparisons (*t*-value) and the percentage contribution of each element per plant organ (50% dissimilarity). The analysis showed the variables that contribute to the dissimilarity. The elements that enhanced the dissimilarity in the leaf compared to the stem were Ni, As, and Cu. V, Cu, and Mn promoted dissimilarity in the leaf compared to the root–rhizome. Finally, V, Mn, and Zn contributed to the dissimilarity in the stem compared to the root–rhizome

### 3.20. Plant Indexes

#### 3.20.1. Bioconcentration Factor (BCF)

**Figure 8:**
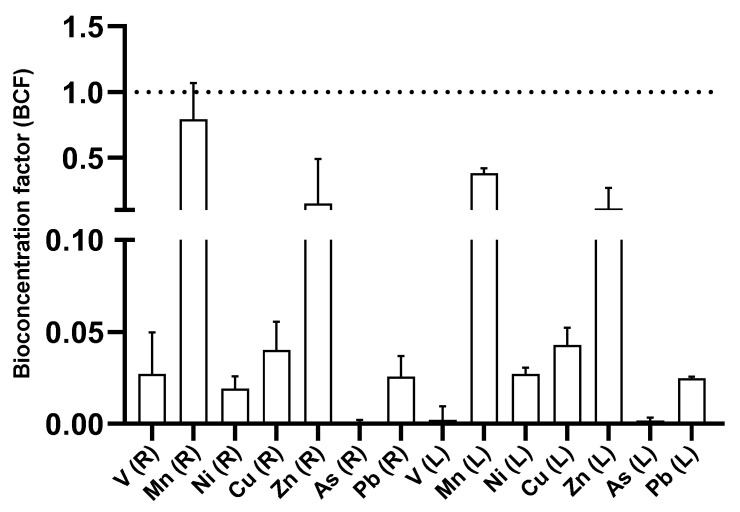
Bioconcentration factor in *P. australis* per organ. (R): root–rhizome, (L): leaf. A BCF greater than 1 indicates that the plant is efficient at accumulating metals.

The BCFs analyzed the efficiency of *P. australis* in accumulating elements in underground (root–rhizome) and aerial (leaf) tissues. The BCF values discovered in the root indicate that the root primarily absorbs the following elements in descending order: Mn, Zn, Cu, Pb, V, and As. The stem and leaf values indicated the descending order of element accumulation: Mn, Zn, Cu, Ni, Pb, V, and As. The rhizome–root and leaf presented values below 1 in the BCF; only in the case of Mn in the root–rhizome did the BCF exceed the value of 1. These low values may be becase this study was conducted in situ and the co-concentrations to which the plant is exposed are low. (Figure 8).

#### 3.20.2. Translocation Factor (TF)

**Figure 9:**
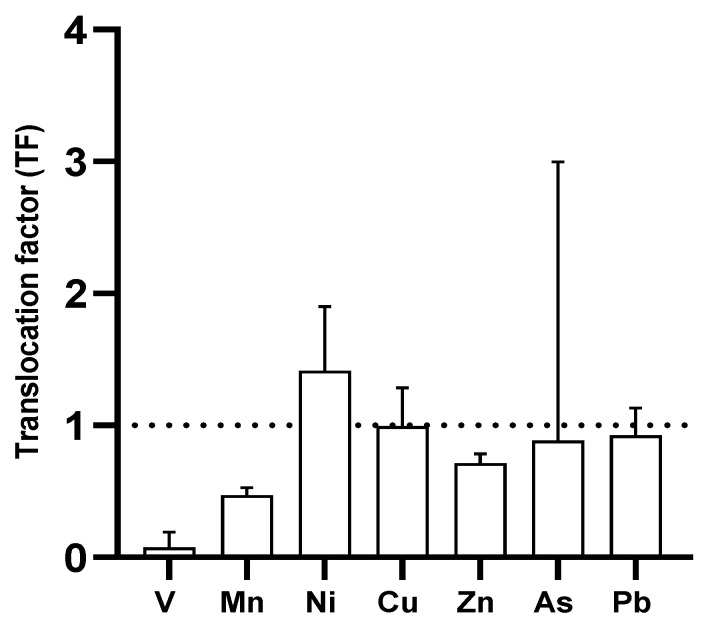
Translocation factor (TF) in *P. australis* by elements. A TF higher than 1 indicates that the plant accumulates metals in its aerial parts.

The TF showed that V, Mn, Zn, As, and Pb obtained a low translocation factor (TF < 1), and Ni and Cu obtained TF values higher than 1 (Figure 9).

### 3.21. Chemical Speciation

The ChemEQL-3.1 software analyzed the chemical speciation of the four sampling seasons and the surface and interstitial water. As (0.004 mg/Kg), Cd (0.0008 mg/Kg), and Pb (0.004 mg/Kg) were mostly found in water samples from the Valles River and different seasons as HAsO_4_^−^, CdCl^+^, CdSO_4_, PbOH^+^, and PbSO_4_. The main chemical forms of As (0.002 mg/Kg), Cd (0.001 mg/Kg), and Pb (0.006 mg/Kg) found in the Valles River’s interstitial water and sediment were CdOH^+^, PbOH^+^, and HAsO_4_^−^, depending on the time of year.

## 4. Discussion

In surface water, univariate and multivariate analyses showed that there were significant differences (*p* < 0.05) with an increase in the concentration of sulfates (13.95–30.72 mg/L) at site 1 during spring and winter, although an increase in concentration was observed that did not exceed the permissible limit (PL = 400 mg/L); chlorides (3.22–28.79 mg/L) at site 2 during spring and summer exceeding the PL = 0.2–1.5 mg/L established by national legislation [31]; TDS (0.040–0.10 µg/L) at site 3 during spring and autumn, for TDS in water there are no PL values in Mexican legislation; and an increase in pH (7.3–7.8) was also observed at site 1 during autumn without exceeding the PL = 6.5–8.5. For other variables, there were no significant differences (*p* < 0.05) by site. However, there were significant differences (*p* < 0.05) by season, with TH concentration going up in the spring and summer and EC (0.08–0.11 mS) going up in the fall. There are no PL values in Mexican law for EC in water. The patterns by site and season observed in the multivariate analysis were consistent with those of the univariate analysis.

Some physicochemical properties of the interstitial water were significantly different (*p* < 0.05). For example, the pH rose from 6.5 to 7.3 at sampling site 1 in the fall. At sampling sites 2 and 3, the concentrations of chlorides rose from 1.0 to 17.0 mmol L^−1^ and 1.2 to 18.0 mmol L^−1^ during winter, spring, and summer. At sampling site 3, the concentrations of sulfates rose from 7.6 to 26.1 mmol L^−1^ during winter, spring, and summer. The EC did not show significant differences by sampling site, but it did show differences by season (*p* < 0.05), during winter and spring in a range of 0.08–0.09 mS and 0.2–0.5 mS during autumn.

Inorganic chemicals and fertilizers found in industrial and urban wastewater discharges could account for the levels of chlorides and sulfates in water. The results agree with the findings observed in Nigeria [32], China [33], South Africa, and Mozambique [34]. Higher amounts of chlorides (789.0 mg/L), sulfates (374.9 mg/L), and elements (7.33 Cd, 11.4 Mn, and 19.6 mg/kg Zn) were found in wastewater from a sugar mill [35]. Torres-Martínez et al. [36] showed that the main sources of sulphate and chloride contamination in groundwater in Monterrey, Mexico, are due to urban and industrial wastewater and atmospheric deposition. Additionally, sewage, household, and animal waste pollute the water, causing changes in pH, TDS, HT, sulfates, and chlorides [37]. On the other hand, surface water evaporation and high carbonate and silicate concentrations can cause TDS levels to rise [38]. Agricultural practices, particularly the application of pesticides, may contribute to the observed pH alterations [39]. The above suggests that pollution in the Rio Valles basin may be mainly due to urban wastewater, agricultural waste, industrial waste mainly from the sugar industry, burning of fossil fuels, and leaching and/or discharge of agrochemicals.

The PTE found in surface waters that showed a significant difference (*p* < 0.05) was As (9.4 × 10^−3^–0.052 mg/kg) at sampling site 1 during winter. According to the official Mexican regulation, the PL for arsenic in drinking water is 0.025 mg/L [31]. This indicates that some points at sampling site 1 exceed the PL, posing a risk to public health. This is because the water operator at this sampling site collects water for purification and distribution to the region, as well as the local ecosystem. Arsenic is a carcinogen that causes several diseases (cardiovascular effects, pulmonary, immunological, endocrine disorders, effects on reproductive health, and neurological disorders) and induces damage to human cells and genetic material [40]. There was an increase in Pb concentration at sampling sites 2 (0.001–0.01 mg/kg) and site 3 (0.002–0.012 mg/L) during autumn. Since the PL is 0.01 mg/kg, the Pb levels at site 3 exceeded the exposure limit value, which poses a potential health risk. Because Pb is an analogue of Ca, it can absorb into the body when there is a calcium (Ca) deficiency, disrupting the central nervous system and damaging the kidneys and brain. Pb accumulates in the brain, liver, kidneys, and bones, which poses a health risk [27].

The findings showed that the PTEs that presented significant differences (*p* < 0.05) in interstitial water were As (0.003–0.01 mg/L) at sampling site 3 during winter, spring, and summer; Cd (0.0005–0.001 mg/L) at sampling site 2 during autumn; and Pb (0.0002–0.009 mg/L) during autumn, with no significant difference between sampling sites.

Anthropogenic activities are responsible for the presence of PTEs like As, Pb, and Cd in water, as well as physicochemical properties like pH, EC, TDS, chlorides, sulfates, and TH at the sampling sites during different seasons. These include agriculture, livestock, inappropriate use of pesticides, vehicle washing, fossil fuel consumption, urbanization, discharge of untreated wastewater, and the sugar industry, a major economic activity in the area. Other studies have identified similar anthropogenic influences as contributors to the contamination of water bodies [29,41,42,43]. Other anthropogenic factors causing contamination with elements (including Pb, Cd, Hg, As, Zn, and Co) include vehicle emissions, livestock activities, plastic waste, and natural phenomena such as geological erosion [41,44,45,46,47].

There were differences between sampling sites and seasons in the sediment, as shown by both the univariate and multivariate analyses. For example, at sampling site 1, the concentrations of Hg (57.6–84.8 mg/kg) and Ni (44.2–207.7 mg/kg) increased in the fall (*p* < 0.05). In the spring and summer seasons, an increase in Cu concentration (71.8–165.2 mg/kg) was observed at sampling site 2 (*p* < 0.05). In the spring season, an increase in As concentration (5.8–44.5 mg/kg) was observed at sampling site 1 (*p* < 0.05). And finally, in the summer, Cd (12.40–23.20 mg/kg) and Mn (113.1–482.3 mg/kg) concentrations were determined at site 1 and Zn (42.6–434.2) and Pb (15.0–45.6) at site 2 (*p* < 0.05). The local sugar mill’s use of fossil fuels, such as fuel oil, is likely to contribute to the elevated concentrations of Hg and Ni at sampling site 1 [48]. In addition, improper disposal of solid waste and untreated wastewater can increase Hg and Ni concentrations in sediments. Moura et al. [49] determined Hg concentrations (dry: 9.94, rainy: 6.14 µg/kg) in sediments of the Jaguaribe River estuary (Brazil), indicating that the main sources causing the presence of Hg in sediments were solid waste disposal and sewage. Saadati et al. [50] recorded the levels (µg/kg) of Hg (2.1–10.75), Cd (3.36–4.03), and Ni (69.41–75.28) in sediment samples from Mousa Bay (northwestern Persian Gulf). Fossil fuel burning, agricultural activities, and the use of phosphate fertilizers contributed to the presence of Hg and Ni in sediments, while urban and industrial wastewater increased the concentration of Cd in the environment. Mercury can come from nonpoint sources such as agricultural runoff (pesticides and fertilizers), storm runoff, urban runoff, and atmospheric deposition [51]. Direct or indirect wastewater discharge from urban and industrial areas, as well as low-scale anthropogenic activities like car washing and fuel burning, are the main sources of these elements. The increase of Zn concentrations in sediments is due to mining extraction and fossil fuel burning [52], and the release of Cd in sediments is due to mining, industrial, and agricultural activities, specifically fertilizers and pesticides [37]. Industrial effluents and domestic wastewater contributed to the high levels of elements (Cd, Zn, Cu, Ni, Pb, and others) in the sediments of the Shima River (southern China) and Kenon Lake (Russia) [53]. In this study, elevated concentrations of V, Ni, and Pb were due to petroleum products (lubricants and fuels) and diesel fuel. The harvest season, spanning from winter to late spring, correlates with the increase in PTEs and physicochemical properties in the Valles River [48]. During the summer and fall, the sugarcane harvest season decreases, and agricultural activities tend to increase. In particular, the sugar industry is a major source of PTEs in local water bodies. For example, Kumar et al. [35] reported the presence of elements (Cd, 0.37 mg/kg; Pb, 0.22 mg/kg; Cr, 0.43 mg/kg; and Mn, 2.05 mg/kg) in natural effluents used by the sugar industry. Naz et al. [54] identified elements (1.2 Pb, 0.8 Ni, 0.2 Cd, and 0.9 Cr mg/kg) in a sugar industry effluent. An effluent from a sugar mill in India recorded elements (6.9, Cd; 9.1, Cr; 13.5, Cu; 15.7, Zn; and 9.7, Mn mg/kg) [28]. The sugar industry, agriculture, and urbanization near the Valles River area negatively affect the environment.

All three sampling sites showed contamination by PTEs. The Pollution Load Index (PLI) values calculated for sampling sites 1 (1.64), 2 (1.81), and 3 (1.77) indicated that all sampling sites were moderately contaminated. The Soil Quality Guideline Quotient Index (SQGQI) for all three sampling sites exceeded the threshold value (1.0). These calculated indices suggest that PTEs present in the sediments may have toxic effects. Lead induces enzyme inhibitions and causes alterations in microbial biomass, posing health risks to plants, animals, and humans [55]. Elements such as Mn, Zn, Cd, Mo, and Pb in high concentrations in sediments are toxic and can threaten living organisms, destabilizing ecosystems [52]. Potentially toxic elements alter the ecosystem services that nature provides to humans [56]. High concentrations of metals such as Cd, Cr, Cu, Ni, and Zn in sediments can induce toxicity, negatively affecting the survival and growth of benthic organisms [53].

In the case of *P. australis*, the univariate and multivariate analyses showed that in autumn, the plant absorbed Cu (3.4–14.7 mg/kg) and Zn (11.6–74.1 mg/kg) at sampling site 3 (*p* < 0.05). In the summer, this macrophyte accumulated Mn (19.6–576.6 mg/kg) at sampling site 1 and Pb (0.4–1.1 mg/kg) at sampling site 3 (*p* < 0.05). In the autumn, *P. australis* accumulated V (0.1–2.0 mg/kg) (*p* < 0.05). And finally, at sampling site 1, *P. australis* accumulated Ni (1.4–4.9 mg/kg) (*p* < 0.05). Univariate and multivariate analyses by organ showed that *P. australis* accumulated Ni (1.8–6.0 mg/kg) in the leaf and concentrations (mg/kg) of Zn (11.4–74.1), V (0.4–2.0), Pb (0.3–1.0), and Mn (37.8–576.6) in the root–rhizome (*p* < 0.05). Metal concentrations increased in autumn and decreased in the rest of the seasons. The main economic activity in the area, the sugar industry, reaches its peak in autumn, contributing to this trend. Therefore, *P. australis* absorbs metals throughout the year. The findings are like those of a study that looked at the phytoremediation potential of *P. australis* over the course of a year and saw that metal concentrations (Cd, Cr, Cu, Fe, Mn, Mo, Ni, Pb, and Zn) rose in the summer and fell in the fall, winter, and spring. *P. australis* is suitable for Mo and Zn phytoremediation because this macrophyte absorbs metals throughout the year [57]. This study showed that *P. australis* accumulated elements at all sampling sites throughout the year, suggesting that this plant removed contaminants from the sediment. These results agree with previous studies. For example, Eid et al. [30] documented, over one year, the accumulation of heavy metals (Cd, Cu, Ni, Pb, and Zn) by young (Cd 45.0; Cu 142.0; Ni 5810.0; Pb 6505.0; and Zn 179.0 mg/kg) and old (Cd 49.0; Cu 129.0; Ni 6814.0; Pb 6654.0; and Zn 183.0 mg/kg) samples of *P. australis* from Lake Burullus (Egypt). The study concluded that this macrophyte can effectively mitigate metal levels in sediments by ex situ phytoremediation. Similarly, Cicero-Fernandez et al. [58] showed that *P. australis* accumulated elements for two years (2012–2013). They concluded that *P. australis* accumulated heavy metals for prolonged periods (0.6–10 years), showing its potential for phytoremediation in sediments contaminated with Ni, Mo, Pb, Cr, Mn, and Zn.

This study selected certain elements (V, Mn, Ni, Cu, Zn, As, and Pb) for the estimation of BCF and TF. The presence of these elements in elevated concentrations could potentially pose a toxicological threat to plants, sediment, and water.

Bioconcentration factor (BCF) values were less than 1 for V (0.03), Mn (0.83), and Zn (0.18) in underground tissues and Ni (0.02) in aerial tissues. However, *P. australis* recorded higher uptake of V, Mn, and Zn in underground tissues and Ni in aerial tissues. The BCF showed that *P. australis* accumulates Cu and Pb in underground and aerial tissues. The BCF values calculated in this study were relatively low due to various physicochemical factors affecting element uptake in a field study context. Previous studies indicated that *P. australis* showed BCF values of 0.32 (Cu), 1.42 (Zn), 0.1–0.3 (Pb), 3.77 (Cd), 0.46 (Ni), and 0.1–0.5 (Cr) [59,60]. This indicates that *P. australis* is a heavy-metal-tolerant species. *P. australis* absorbs elements (Cu, Ba, V, Cl, Sr, and Mn) through the roots, as shown by multivariate analysis. In contrast, the leaves showed an uptake of Rb, Ni, As, and Mg, and the stem showed no significant accumulation of metals. Univariate analysis indicated that leaves contained concentrations (mg/kg) of Rb (4.5–32.9), Ni (1.8–6.0), and Cl (696.7–1857.2). Roots showed elevated concentrations (mg/kg) of V (0.4–2.0), Ba (191.1–836.0), Zn (11.4–74.1), Mn (37.8–576.6), Pb (0.3–1.0), and Sr (25.6–174.6). These results indicate that the root of *P. australis* is the organ with the highest metal uptake capacity, followed by the leaves, while the stems showed minimal accumulation of heavy metals.

The translocation factor (TF) corroborated these results. Elements such as V (0.11), Mn (0.44), Zn (0.67), Pb (0.90), Sr (0.76), and Ba (0.13) presented TF values lower than 1, indicating that roots and rhizomes retained these metals. On the contrary, Ni (1.38), Cu (1.02), and Rb (1.18) presented TF values higher than 1, suggesting their accumulation by *P. australis* in leaves. A TF value lower than 1 indicates that the roots absorbed the metals, implying that rhizofiltration is the mechanism through which *P. australis* absorbs the elements. Previous studies reported that *P. australis* accumulates concentrations of metals such as Cr, Ni, Cu, and Zn in underground parts [61,62,63]. This study supports those findings, reinforcing that rhizofiltration is a key mechanism by which *P. australis* accumulates heavy metals in situ.

In the surface and interstitial water from the Valles River, the PTEs were found in different chemical forms that were affected by physicochemical conditions such as pH, electrical conductivity, total dissolved solids, chlorides, and sulfates. The primary PTEs found in the Valles River were chlorides (CdCl^+^), sulfates (CdSO_4_, PbSO_4_), and hydroxides (PbOH^+^, Cd_2_OH^+^), according to ChemEQL-3.1 software modeling. Duan et al. [63] demonstrated that heavy metals can exist in water in various chemical forms (cations, anions, and hydroxide complexes), depending on dissolved oxygen, pH, and organic matter content. Similarly, Teramoto et al. [64] found that the chemical speciation of elements in the Paraopeba River (Brazil) included hydroxides, sulfates, cations, anions, and carbonates. These findings align with those observed in this study. *P. australis* probably absorbed the elements in the chemical forms predicted by the ChemEQL-3.1 software. Other studies evaluated the capacity of *P. australis* to accumulate metals. However, these studies did not focus on the chemical form of the metals and analyzed only their ionic form [65,66,67].

The phytoextraction capacity of *P. australis* presents an opportunity to accumulate heavy metals derived from agricultural activities and the sugar industry, and urbanization along the Valles River establishes barriers or corridors. A management plan for utilizing *P. australis* is essential to minimize anthropogenic activities and preserve water quality in the Valles River.

Some limitations of this study were as follows: (1) Short work duration (one year). Longer-duration studies (more than 3 years) could gather data on the mobilization of heavy metals in water, sediment, and plants. This extended period would yield insights into the long-term accumulation of PTEs by macrophytes. (2) Future research should consider additional sampling sites along the Valles River to enhance the breadth and accuracy of findings.

Many studies have not assessed the effects of heavy metal accumulation by macrophytes, considering the three matrices (water, sediment, and plant). Therefore, further studies should include the association of water, sediment, and plants in heavy metal absorption by macrophytes.

## 5. Conclusions

For one year, this study quantified the concentration of PTEs (As, Pb, Cd, and Hg) and physicochemical properties (pH, total hardness, electrical conductivity, total dissolved solids, chlorides, and sulfates) in surface and interstitial water from three sampling sites in the Valles River. Sampling site 1 exhibited arsenic levels exceeding permissible limits set by Mexican legislation, posing a significant environmental risk. Agricultural practices, industrial activities, and urbanization contributed to levels of Hg, Mn, Ni, Zn, Pb, V, Cu, Cr, and Cd recorded in the sediments from the three sampling sites. These sediments were classified as moderate to extremely contaminated during winter, spring, and occasionally summer, showing high levels of PTEs primarily linked to the sugar industry, a major economic driver in the region. According to the Sediment Quality Guideline Quotient Index (SQGQI), these PTEs may lead to toxic effects and health risks for aquatic life and local communities. *P. australis* accumulated elements (Mn, Rb, V, Sr, Cu, Zn, Pb, Ni, and As) from river sediments, actively contributing to the remediation of contaminated sites for one year. The plant accumulated these elements mainly in its root–rhizomes through a process known as rhizofiltration. This study underscores the urgent need for continued monitoring and management strategies to address heavy metal contamination in the Valles River. Anthropogenic activities significantly contribute to water and sediment pollution, making collaboration between local authorities, industries, and communities essential for the implementation of sustainable practices. Furthermore, the results argue for the use of *P. australis* as a cost-effective and environmentally friendly phytoremediation method. Future research should focus on optimizing the application of this plant in various contaminated environments and exploring its potential to restore ecological balance and improve public health outcomes in affected regions.

## Figures and Tables

**Table 2:** Cumulative percentage of physicochemical properties and PTEs in water by SIMPER analysis by site.

Site	*t*-Value (Cumulative Percentage)
Site 1 vs. Site 2	2.2, (50%) * (As, Sulfates, °C, DMg)
Site 1 vs. Site 3	2.7, (50%) * (TDS, Cd, pH, Pb, As)
Site 2 vs. Site 3	1.8, (50%) (TDS, Cd, CE, Pb)

PERMANOVA analysis *t*-value, (*) significant differences (*p* < 0.01).

**Table 4:** Cumulative percentage of physicochemical properties and PTEs in interstitial water by SIMPER analysis by site.

Site	*t*-Value (Cumulative Percentage)
Site 1 vs. Site 2	1.8, (50%) * (pH, Chlorides, Cd)
Site 1 vs. Site 3	4.0, (50%) * (Sulfates, pH, Cd)
Site 2 vs. Site 3	2.3, (50%) (Chlorides, Pb, EC)

PERMANOVA analysis *t*-value, (*) significant differences (*p* < 0.01).

**Table 5:** Cumulative percentage of physicochemical properties and PTEs in interstitial water by SIMPER analysis by season of the year.

Season	*t*-Value (Cumulative Percentage)
Autumn vs. winter	3.9, (50%) * (EC, Pb, Cd)
Autumn vs. spring	3.6, (50%) * (EC, Cd, Pb)
Autumn vs. summer	3.6, (50%) (EC, Chlorides, pH)
Winter vs. spring	1.4, (50%) * (Pb, Cd, Sulfates)
Winter vs. summer	2.0, (50%) * (Chlorides, As, pH)
Spring vs. summer	1.5, (50%) * (Chlorides, As, Sulfates)

PERMANOVA analysis *t*-value, (*) significant differences (*p* < 0.01).

**Table 6:** Cumulative percentage of physicochemical properties and elements in sediment using SIMPER analysis by site.

Site	*t*-Value (Cumulative Percentage)
Site 1 vs. Site 2	3.1, (50%) * (Zn, Pb, V, Cu, Hg, Mn)
Site 1 vs. Site 3	2.8, (50%) * (Ni, Pb, Mn, Cr, Cd)
Site 2 vs. Site 3	1.2, (50%) (V, Cu, Zn, As, Cd)

PERMANOVA analyses *t*-value, (*) significant differences (*p* < 0.01).

**Table 8:** Geoaccumulation index (I_geo_) of elements in sediments collected at three sites in the Valles River, Cd Valles, S.L.P. Pb, <0–0: not contaminated, 0–1: not contaminated to moderately contaminated, 1–2: moderately contaminated, 2–3: moderately to heavily contaminated, 3–4: heavily contaminated, 4–5: heavily to extremely contaminated, >5: extremely contaminated.

Site	V	Cr	Mn	Ni	Cu	Zn	As	Cd	Hg	Pb
Site 1	0.14	1.17	0.11	2.79	1.54	1.46	5.55	38.92	3514.30	0.85
Site 2	0.18	1.15	0.09	2.48	2.17	6.93	5.12	39.36	3027.22	1.54
Site 3	0.16	1.23	0.09	2.38	2.00	5.38	4.97	40.62	3162.56	1.43

**Table 9:** Cumulative percentage of EFA in plant through SIMPER analysis by site.

Site	*t*-Value (Cumulative Percentage)
Site 1 vs. Site 2	5.4, (50%) * (Mn, V, Ni)
Site 1 vs. Site 3	10.0, (50%) * (Cu, Zn, Pb)
Site 2 vs. Site 3	9.2, (50%) * (Cu, Zn, As)

PERMANOVA analysis *t*-value, (*) significant differences (*p* < 0.01).

**Table 10:** Accumulated percentage of PTEs in plants through SIMPER analysis by season.

Season	*t*-Value (Cumulative Percentage)
Autumn vs. winter	6.1, (50%) * (Zn, V, Pb)
Autumn vs. spring	7.2, (50%) * (Cu, Zn, Ni,)
Autumn vs. summer	9.6, (50%) * (Zn, Mn, As)
Winter vs. spring	5.6, (50%) * (Cu, Ni, Pb)
Winter vs. summer	6.1, (50%) * (Mn, As, Pb)
Spring vs. summer	5.6, (50%) * (Cu, Mn, Ni)

PERMANOVA analysis *t*-value, (*) significant differences (*p* < 0.01).

**Table 11:** Accumulated percentage of elements in plants through SIMPER analysis by organ.

Organ	*t*-Value (Cumulative Percentage)
Leaf vs. stem	6.8, (50%) * (Ni, As, Cu)
Leaf vs. root–rhizome	12.3, (50%) * (V, Cu, Mn)
Stem vs. root–rhizome	15.3, (50%) * (V, Mn, Zn)

PERMANOVA analysis *t*-value, (*) significant differences (*p* < 0.01).

## Data Availability

The data are contained within this article.
